# The surgical outcomes and risk factors of giant hepatic haemangiomas: a single centre experience

**DOI:** 10.1186/s12893-022-01721-w

**Published:** 2022-07-17

**Authors:** Zhitao Dong, Kunpeng Fang, Chengjun Sui, Junwu Guo, Binghua Dai, Li Geng, Jiamei Yang

**Affiliations:** Department of Special Treatment, Shanghai Eastern Hepatobiliary Surgery Hospital, The 15th floor, No.225 Changhai Road, YangPu district, Shang Hai, 200438 China

**Keywords:** Liver, Liver resection, Hepatic cavernous haemangioma

## Abstract

**Objective:**

To evaluate the safety of performing surgery on cavernous haemangiomas in the liver larger than 10 cm and establish preoperative predictors of intraoperative blood transfusion and morbidity.

**Methods:**

A total of 373 patients with haemangiomas larger than 10 cm who underwent surgery in our hospital were retrospectively analysed. According to tumour diameter, the patients were divided into a giant haemangioma (GH) group (241 cases) (10 cm ≤ diameter < 15 cm) and an enormous haemangioma (EH) group (132 cases) (diameter ≥ 15 cm). Clinical parameters were then compared between the two groups.

**Results:**

Compared with the GH group, the EH group had higher rates of leukopenia (10.6% vs. 4.5%), anaemia (26.5% vs. 15.7%), and thrombocytopenia (13.6% vs. 6.2%). The occlusion time in the EH group was longer than that in the GH group (26.33 ± 14.10 min vs. 31.85 ± 20.09 min, P < 0.01). The blood loss and blood transfusion in the EH group were greater than those in the GH group (P < 0.05). Moreover, the morbidity in the EH group was higher than that in the GH group (17.4% vs. 9.13%, P < 0.05). According to the results of the multivariable analysis, the operation time and size of the haemangioma may be independent risk factors for blood transfusion (P < 0.05). Additionally, the size of the haemangioma may be an independent risk factor associated with complications (P < 0.05).

**Conclusion:**

Enormous haemangioma is more likely to cause haematologic abnormalities than giant hepatic haemangioma. The risks of the operation and postoperative complications of enormous haemangioma are higher than those of giant hepatic haemangioma.

## Introduction

Hepatic cavernous haemangioma is the most common benign tumour in the liver, with a prevalence rate of 3% to 20% [1]. It can occur at any age, and most cavernous haemangiomas in the liver are found in females in their 40s or 50s. The majority of hepatic haemangiomas are asymptomatic and incidentally diagnosed.

Hepatic haemangiomas larger than 5 cm were previously considered giant hepatic haemangiomas. However, recent studies have suggested that haemangiomas with a diameter greater than 10 cm should be considered giant hepatic haemangiomas [2], which is more consistent with the tumour characteristics and requirements for clinical diagnosis and treatment [3, 4].

Although recent advances in surgical techniques have allowed hepatic haemangioma resection to be performed safely, controversies still exist regarding the management of haemangiomas. The average size of reported lesions was generally less than 10 cm. Few reports have described haemangiomas ≥ 10 cm in size. Data comparing the results of different surgical methods are limited, especially for methods involving enucleation with liver resection in haemangiomas larger than 10 cm. In addition, haemorrhage continues to be a major concern during the perioperative period, particularly in haemangiomas larger than 10 cm, due to the risk of dissection and vascular damage [2, 5]. Moreover, haemangioma with a diameter ≥ 10 cm is more likely to compress peripheral blood vessels and gastrointestinal organs, resulting in abnormal liver blood flow and gastrointestinal discomfort [5]. Surgical treatment can effectively relieve the compression symptoms of haemangiomas on peripheral blood vessels and gastrointestinal organs.

In this large retrospective study, we report our experience managing patients with hepatic haemangiomas larger than 10 cm. The clinical characteristics and haematological changes in haemangiomas with different diameters were compared and analysed. The risk factors for haemorrhage and postoperative complications of giant haemangiomas (larger than 10 cm) were analysed, and the effect of surgical treatment on patients' symptoms and haematological indicators.

## Patients and methods

The protocol used in this study was consistent with the principles of the 2013 Helsinki Declaration and approved by the ethics committee for clinical trials of the Eastern Hepatobiliary Surgery Hospital. Informed consent was obtained from all patients for the diagnostic and therapeutic procedures. All resected specimens were verified by histopathological examination.

### Patients

Between January 2010 and September 2015, 373 patients with hepatic haemangiomas (larger than 10 cm) underwent surgical resection at the Eastern Hepatobiliary Surgery Hospital.

The inclusion criteria for this study were a single lesion larger than 10 cm or multiple masses with at least one larger than 10 cm along with any of the following surgical indications: persistent abdominal distention, abdominal pain, postprandial fullness and discomfort, and anxiety for rapid growth (annual growth greater than 2 cm) [6–8]. The exclusion criteria consisted of patients with cirrhosis or another significant comorbidity who refused to participate.

Patient records were retrospectively analysed. The collected data included the following: patient information, characteristics of the haemangiomas, laboratory tests, surgical data (operative procedure, surgical time, blood loss, blood transfusion, and occlusion time), hospital stay, postoperative complications, and mortality.

Patients were divided into the following two groups according to the diameter of the hepatic haemangioma: an enormous haemangioma (EH) group (size ≥ 15 cm) and a giant haemangioma (GH) group (10 cm ≤ size < 15 cm). Then, the two groups were compared regarding various indexes.

### Methods

Most hepatic haemangiomas can be diagnosed preoperatively with imaging techniques such as computed tomography (CT) and magnetic resonance imaging (MRI), and MRI is the preferred method for diagnosing hepatic haemangioma. PET-CT (positron emission tomography-computed tomography) is used to further clarify the diagnosis in some patients whose diagnosis is unclear (e.g., hepatic adenomas, hepatic endothelioma, hepatic cystadenocarcinoma). Before surgical resection, it is important to exclude other reasons for abdominal symptoms, especially when the patient's symptoms are vague or indefinite, such as gallstones, inflammatory bowel diseases, gastroesophageal reflux or peptic ulcer disease.

When the patient's abdominal discomfort symptoms are inconsistent with the diameter and location of the haemangioma, gastroscopy and enteroscopy are routinely performed. All excised specimens were subjected to routine pathology examinations.

### Surgical techniques

Hepatectomy and haemangioma enucleation are the most common surgical procedures for giant hepatic haemangioma.

Liver resections included sectionectomy, hemihepatectomy, and trisectionectomy. Hepatic haemangioma enucleation removes the haemangioma without damaging the surrounding normal liver tissue.

Many studies have confirmed that hepatectomy and haemangioma enucleation are safe and feasible if the indications are properly controlled [2, 5, 7, 9, 10].

The surgical approach depends on the location and diameter of the tumour, its relationship with the peripheral vasculature and the volume of the residual liver. When the hepatic haemangioma occupies half the liver or the left lateral lobe, liver resection is performed, similar to the approach for deep cavernous haemangiomas in the liver or those with multiple lesions [11].

In patients with multiple hepatic haemangiomas, we attempted to remove all lesions at once, starting with the largest lesion. However, if the residual liver volume is insufficient and the surgical risk is too high, small hepatic haemangiomas should not be resected (< 5 cm). Intraoperative ultrasonography was routinely used during the operation. The Pringle manoeuvre was routinely used during operations in cycles of 15/5 min of clamp/unclamp time. All intraoperative and postoperative transfusion indications were in accordance with ASA guidelines [12]. Early postoperative enteral nutrition support was provided after the patient's gastrointestinal function was restored. Routine blood tests were performed on the first, third, and seventh postoperative days, and additional blood tests were performed according to the patient’s situation.

All patients provided informed consent to the diagnosis and treatment procedures. The study was approved by the institutional Ethics Review Board of Eastern Hepatobiliary Surgery Hospital.

### Follow-up

Patient follow-up included clinical examinations and ultrasound examinations performed every 6 months within 2 years after the operation and each year thereafter. The patients’ report of symptom relief was also assessed.

### Statistical analysis

Continuous variables are shown as the means ± standard deviations and were compared with Student’s t test. The chi-square or Fisher’s exact test was used to compare categorical variables. The Mann–Whitney U test was applied for nonparametric variables. Independent variables that affected blood transfusion and morbidity were determined by logistic regression analysis. A value of P < 0.05 was considered statistically significant. SPSS (version 21.0; IBM Corp, Armonk, NY) software was used for the statistical analyses.

## Results

In this study, surgery was performed in 373 patients with cavernous haemangiomas of the liver (size ≥ 10 cm). The patient characteristics are listed in Table 1. A total of 9 patients met the inclusion criteria of this study but were excluded after meeting one of the exclusion criteria, including 2 patients with severe hepatitis cirrhosis (decompensated), 1 patient with severe coronary heart disease, 1 patient with right heart failure, 2 patients with severe diabetes, 1 patient with renal failure, and 2 patients declining to participate.

**Table 1:** Comparison of general characteristics and laboratory tests of different diameter groups

Demographics/characteristics		GH group (n = 241)	EH group (n = 132)	P-value
Sex (male/female)		81/160	45/87	0.984
Median age		46	45	
Reason for evaluation				
Symptomatic		59 (24.5%)	37 (28.0%)	
Upper abdominal discomfort		29	14	
Indigestion		7	3	
Right or left quadrant pain		22	17	
Abdominal mass		1	3	
Rapid growth		182 (75.5%)	95 (72.0%)	
ASA grading		1.45 + 0.49	1.47 + 0.51	0.317
Preoperative lab tests	Normal range			
Leukocyte (× 10^9^/L)	4.0–10.0	5.2 ± 1.1	5.0 ± 0.8	0.065
Leukopenia		11 (4.5%)	14 (10.6%)	0.043
Hemoglobin (g/L)	Female 115–150 Male 120–155	130.24 ± 15.2	127.17 ± 16.97	0.195
Anemia		38 (15.8%)	35 (26.5%)	0.018
Thrombocyte (× 10^9^/L)	110–320	202.56 ± 59.06	183.77 ± 50.64	0.550
Thrombocytopenia		15 (6.2%)	18 (13.6%)	0.026
Pancytopenia cases		1 (0.4%)	2 (1.5%)	0.590
ALT (U/L)	0–40	31.23 ± 38.40	27.76 ± 36.09	0.679
Increased ALT cases		48 (19.9%)	20 (15.15%)	0.317
AST (U/L)	0–42	28.01 ± 49.37	24.52 ± 38.68	0.194
Increased AST cases		19 (7.8%)	13 (9.8%)	0.649
ALB (g /L)	0–52	43.41 ± 3.07	43.61 ± 3.55	0.216
Total bilirubin (mmol/L)	5.0–21.0	12.31 ± 4.43	14.29 ± 5.13	0.102
Hyperbilirubinemia cases		10 (4.14%)	17 (12.8%)	0.003
PT (s)	10–13	11.27 ± 0.75	11.54 ± 0.83	0.067
High prothrombin time cases		1 (0.4%)	6 (4.5%)	0.019

*ALT* alanine aminotransferase, *AST* aspartate aminotransferase, *ALB* albumin

The median patient age was 46 (range 25–66) years old. A total of 247 (66.2%) females and 126 (33.8%) males were included in our study. A total of 286 patients (76.67%) were asymptomatic but exhibited rapid tumour growth. The symptoms of the other patients included abdominal pain (n = 43), epigastric discomfort (n = 32), dyspepsia/indigestion (n = 9), and abdominal mass (n = 3). No spontaneous rupture was found in our study.

All patients underwent conventional preoperative ultrasonography, and MRI or CT was performed in the majority of the patients. In 368 (98.66%) patients, haemangioma was diagnosed by 2 imaging techniques, and haemangioma was diagnosed in 5 (1.34%) patients by 3 imaging techniques (MRI, CT, PET-CT). No patient was suspected of having cancer. Percutaneous or intraoperative biopsies were not used for diagnosis. The average diameter of the haemangiomas was 14.33 ± 3.93 (range 10–40) cm. The tumour was located in the right lobe of the liver in 190 (50.9%) patients, the left lobe of the liver in 100 (26.8%) patients and bilaterally in the liver in 83 (22.3%) patients. A total of 289 (77.5%) patients had a single lesion, and 84 (22.5%) patients had multiple lesions.

A total of 173 patients (71.8%) in the GH group and 50 patients (37.9%) in the EH group underwent haemangioma enucleation. Sixty-eight patients (28.2%) in the GH group underwent liver resection, including 24 patients who underwent hepatic sectionectomy, 37 who underwent hemihepatectomy, and 7 who underwent hepatic trilobectomy. Eighty-two patients (62.1%) in the EH group underwent liver resection, including 17 patients who underwent hepatic sectionectomy, 53 who underwent hemihepatectomy, and 12 who underwent hepatic trilobectomy. All specimens were pathologically identified as hepatic cavernous haemangioma.

The patients in the EH group had higher rates of leukopenia (10.6% vs. 4.5%), anaemia (26.5% vs. 15.7%), and thrombocytopenia (13.6% vs. 6.2%) than those observed in the GH group. No differences were detected in the serum alanine aminotransferase (ALT), albumin (ALB), aspartate aminotransferase (AST), or total bilirubin (TB) levels or the prothrombin time (PT) between the two groups (P > 0.05). However, the patients in the EH group were more likely to have hyperbilirubinemia and a prolonged PT (P < 0.05) (Table 1).

All postoperative complications were recorded and summarised according to the Clavien–Dindo classification [1, 13].

All resected specimens were confirmed by histopathological examination.

### Outcomes in patients with different haemangioma diameters

The preoperative variables were similar between the GH and EH groups. No statistically significant difference was observed in the operation time, the number of resected lesions, the use of the Pringle manoeuvre, postsurgical hospital stay or mortality between the two groups. Hepatectomy was more common in the EH group, while haemangioma enucleation was more common in the GH group (P < 0.01). The occlusion time in the EH group was longer than that in the GH group (P < 0.01). The blood loss and blood transfusion in the EH group were more significant than those in the GH group (P < 0.05, P < 0.01). Moreover, the morbidity in the EH group was higher than that in the GH group (all P < 0.05) (Table 2).

**Table 2:** Comparison of operative characteristics of GH and EH groups

Demographics/characteristics	GH group (n = 241)	EH group (n = 132)	P-value
Operative time (min)	197.26 ± 37.33	254.85 ± 46.22	0.081
Operative procedure			
Enucleation	173 (71.8%)	50 (37.9%)	
Liver resection	68 (28.2%)	82 (62.1%)	< 0.01
Hepatic Trilobectomy	7	12	
Hemihepatectomy	37	53	
Hepatic sectionectomy	24	17	
Use of Pringle maneuver	217 (90.0%)	124 (93.9%)	0.227
Occlusion time (min)	26.33 + 14.10	31.85 + 20.09	< 0.01
Blood loss (ml)	608.30 ± 757.76	1378.03 ± 1671.37	< 0.05
Blood transfusion cases	82 (34.0%)	49 (37.1%)	< 0.01
Morbidity			
N	219 (90.9%)	109 (82.6%)	0.029
Y	22 (9.1%)	23 (17.4%)	
Mortality	1	1	0.583
Postoperative stay (d)	10.73 + 3.34	12.37 + 3.23	0.548

### Outcomes of different surgical treatments

The surgical procedures included hepatectomy and haemangioma enucleation. Within the GH group, there was no significant difference in AST, haemoglobin, thrombocytes, the use of the Pringle manoeuvre, blood transfusion or postoperative hospital stay between the liver resection subgroup (LR group) and the enucleation subgroup (EN group). ALT and TB levels on the 1st postoperative day in the liver resection subgroup were higher than those in the enucleation subgroup (P < 0.05). Meanwhile, the operative time and occlusion time were longer in the liver resection subgroup than in the enucleation group (P < 0.05), while morbidity and mortality were similar between these subgroups.

In the EH group, the ALT level on the 7th postoperative day was higher in the liver resection subgroup than in the enucleation subgroup (P < 0.05). However, no significant difference was found for any other characteristic. Morbidity and mortality were also similar between the two subgroups (Table 3).

**Table 3:** Comparison of laboratory tests between the GH and EH groups

Demographics/characteristics	GH group (n = 241)		P-value	EH group (n = 132)		P-value
	EN (n = 173)	LR (n = 68)		EN (n = 50)	LR (n = 82)	
AST (U/L)						
Pre-operation	26.35 ± 38.99	32.26 ± 69.29	0.054	19.72 ± 20.16	27.44 ± 46.38	0.128
1st postoperative day	606.88 ± 478.53	360.99 ± 433.97	0.134	497.43 ± 398.41	368.54 ± 407.14	0.312
7th postoperative day	55.32 ± 44.85	42.66 ± 30.41	0.179	70.96 ± 118.40	44.64 ± 34.00	0.007
ALT (U/L)						
Pre-operation	31.14 ± 39.56	31.46 ± 35.58	0.555	26.42 ± 26.79	28.59 ± 40.88	0.445
1st postoperative day	622.08 ± 516.99	310.49 ± 388.49	0.007	459.9 ± 388.27	310.41 ± 280.93	0.227
7th postoperative day	175.19 ± 115.33	94.02 ± 82.03	< 0.01	117.86 ± 82.63	102.04 ± 74.39	0.358
Total bilirubin (μmol/L)						
Pre-operation	12.42 ± 4.40	12.03 ± 4.51	0.369	12.99 ± 4.13	15.09 ± 5.52	0.051
1st postoperative day	19.71 ± 10.24	30.00 ± 59.96	0.001	24.88 ± 15.03	33.32 ± 31.72	0.074
7th postoperative day	15.55 ± 10.86	17.59 ± 20.25	0.076	17.52 ± 11.80	21.15 ± 15.40	0.093
Hemoglobin (g/L)						
Pre-operation	131.68 ± 14.20	126.60 ± 17.25	0.619	128.98 ± 13.94	126.07 ± 18.58	0.055
1st postoperative day	118.60 ± 16.67	118.35 ± 16.73	0.871	119.06 ± 13.10	116.43 ± 17.01	0.067
7th postoperative day	106.42 ± 15.78	108.71 ± 14.09	0.338	107.48 ± 12.85	109.77 ± 20.39	0.058
Thrombocyte (× 10^9^/L)						
Pre-operation	208.86 ± 62.84	186.54 ± 44.70	0.094	189.84 ± 37.80	180.07 ± 56.98	0.054
1st postoperative day	182.64 ± 59.05	162.12 ± 47.89	0.261	156.00 ± 44.17	153.37 ± 55.91	0.101
7th postoperative day	231.62 ± 76.98	217.63 ± 54.58	0.362	218.18 ± 62.63	217.02 ± 79.45	0.100
Operative time (h)	195.31 ± 32.54	202.21 ± 47.31	0.037	245.00 ± 39.14	260.85 ± 49.32	0.122
Use of Pringle maneuver	158	59	0.380	46	78	0.723
Occlusion timey (min)	25.71 ± 11.68	27.91 ± 18.94	0.003	30.32 ± 16.50	32.78 ± 22.04	0.177
Blood loss (mL)	531.50 ± 499.45	803.68 ± 1167.26	< 0.01	1225.00 ± 1781.40	1471.34 ± 1604.64	0.679
Blood transfusion cases	58 (33.5%)	24 (35.3%)	0.912	18 (36.0%)	31 (37.8%)	0.982
Morbidity	13	9	0.254	12	11	0.187
Mortality	0	1		1	0	
Postoperative stay (d)	9.38 ± 2.52	11.85 ± 3.81	0.782	11.44 ± 3.51	13.39 ± 5.49	0.586

*EN* enucleation of hepatic hemangioma, *LR* liver resection of hepatic hemangioma

### Analysis of factors related to blood transfusion

The mean blood loss in our study was 678.54 ± 1.045.79 (range 100–9600) mL. One hundred thirty-one (35.12%) patients received a blood transfusion. The average blood transfusion volume was 716.53 ± 1402.61 (range 0–6400) mL.

The results of the univariable analysis of the association between various parameters and blood transfusion are shown in Table 4. The size, location, operation time and operation method used for the haemangioma were significantly correlated with blood transfusion (P < 0.05).

**Table 4:** Univariable and multivariable analysis of risk factors of Blood transfusion

Variable	Untransfused (n = 242)	Transfused (n = 131)	Univariable analysis		Multivariable analysis	
			OR (95%CL)	P-value	OR (95%CL)	P-value
Gender						
Male	78 (32.2%)	48 (36.6%)	1.488 (0.803–2.756)	0.456		
Female	164 (67.8%)	83 (63.4%)				
Age						
≪60	231 (95.5%)	125 (95.4%)	1.284 (0.739–2.231)	0.807		
> 60	11 (4.5%)	6 (4.6%)				
Number						
Solitary	195 (80.6%)	94 (71.8%)	1.757 (0.952–3.241)	0.069		
Multiple	47 (19.4%)	37 (28.2%)				
Location						
Unilateral	189 (78.1%)	101 (77.1%)	1.192 (0.601–2.366)	0.927		
Bilateral	53 (21.9%)	30 (22.9%)				
Size						
GH group	159 (65.7%)	82 (62.6%)	1.857 (1.040–3.315)	< 0.01	0.093 (0.034–0.252)	< 0.05
EH group	83 (34.3%)	49 (37.4%)				
Operative time (min)	193.8 + 25.2	261.6 + 52.1	1.942 (1.121–3.392)	< 0.01	1.091 (1.072–1.111)	< 0.05
Operative method						
Major hepatectomy	67 (27.7%)	54 (41.2%)	1.968 (1.264–3.057)	< 0.01	1.236 (0.673–2.657)	0.596
Minor hepatectomy /enucleation	188 (72.3%)	77 (58.8%)				

Location refers to the location of the largest liver hemangioma for patients with multiple lesion

Major hepatectomy included resection of three or more segments (right hepatectomy, left hepatectomy, extended right hepatectomy, extended left hepatectomy, and any trisegmentectomy

Minor hepatectomy included resection of two or fewer segments and non-anatomic wedge resection according to the classification of Couinaud

Multivariable analysis results suggest that the operation time and size of the haemangioma may be independent risk factors for blood transfusion. (OR 1.091, 95% CI 1.072 to 1.111, P < 0.05, and OR 0.093, 95% CI 0.034 to 0.252, P < 0.05, respectively; Table 4).

### Parameters associated with complications

Complications occurred in 45 patients (12.06%). Pleural effusion was the most common surgical complication in 12 patients (3.22%). Other complications included abdominal cavity effusion in 11 patients (2.95%), abdominal cavity effusion and pleural effusion in 7 patients (1.88%), haemorrhage in 8 patients (2.14%), renal failure in 1 patient (0.27%), poor wound healing in 1 patient (0.27%), acute renal failure in 1 patient (0.27%), jaundice in 1 patient (0.27%), and bile leakage in 1 patient (0.27%). One patient died due to respiratory failure, and another patient died due to intra-abdominal infection (0.54%). We reclassified the patients according to the Clavien–Dindo classification method as follows: Grade I (17 cases) (4.56%), Grade II (6 cases) (1.61%), Grade IIIa (17 cases) (4.56%), Grade IIIb (2 cases) (0.54%), Grade IVa (1 case) (0.27%), and Grade V (2 cases) (0.54%). The patients with grade IIIa (17 cases) were complicated with pleural and peritoneal effusion and cured after puncture treatment; The patients with grade IIIb (2 cases) were complicated with postoperative jaundice and bile leakage, which were cured after ERCP treatment; The patients with grade IVa (1 case) was complicated with renal failure, which was recovered after conservative treatment; The patients with grade V (2 cases) died of abdominal bleeding and abdominal infection respectively.

The correlations between parameters and complications are shown in Table 5. A larger tumour size, greater blood loss and a longer operation time were significantly more common in patients with complications (P < 0.05). In the multivariable analysis, the results suggested that the size of the haemangioma may be an independent risk factor associated with complications (OR 2.101, 95% CI 1.121 to 3.936, P = 0.021).

**Table 5:** Univariable and multivariable analysis of risk factors for postoperative morbidity

Variable	No morbidity (n = 328)	Morbidity (n = 45)	Univariable analysis		Multivariate analysis	
			OR (95% CL)	P-value	OR (95% CL)	P-value
Gender (male/female)	110/218	16/29	1.223 (0.637–2.145)	0.920		
Age	46.32 ± 8.12	44.62 ± 8.98	1.630 (0.917–2.896)	0.344		
ASA grading	1.46 + 0.51	1.47 + 0.50	1.363 (0.731–2.926)	0.818		
Number						
Solitary	256 (78.0%)	33 (73.3%)	1.489 (0.842–2.631)	0.603		
Multiple	72 (22.0%)	12 (26.7%)				
Operative time (min)	217.56 ± 48.53	218.22 ± 53.74	1.974 (1.080–3.608)	0.085		
Operative method						
Major hepatectomy	118 (36.0%)	14 (31.1%)	1.277 (0.667–2.483)	0.473		
Minor hepatectomy/enucleation	210 (64.0%)	31 (68.9%)				
Blood transfusion						
No	222 (67.7%)	20 (44.4%)	2.599 (1.409–4.794)	0.004	0.909 (0.455–1.819)	0.788
Yes	106 (32.3%)	25 (55.6%)				
Size						
GH group	219 (66.8%)	22 (48.9%)	2.112 (1.145–3.896)	0.029	2.101 (1.121–3.936)	0.021
EH group	109 (33.2%)	23 (51.1%)				

Major hepatectomy included resection of three or more segments (right hepatectomy, left hepatectomy, extended right hepatectomy, extended left hepatectomy, and any trisegmentectomy

Minor hepatectomy included resection of two or fewer segments and non-anatomic wedge resection according to the classification of Couinaud

### Follow-up

Clinical follow-up data were available for 323 patients. The average follow-up period was 43 (range 7–98) months. Eighty-seven patients were symptomatic preoperatively. The postoperative symptoms of 84 patients (96.55%) were completely or significantly relieved, but three patients still had persistent postoperative symptoms. Two patients had persistent upper abdominal discomfort due to a history of erosive gastritis and duodenal ulcers. Another patient developed abdominal discomfort because of hepatitis.

Haematologic abnormalities were also corrected in most patients after the operation. Three patients (3/373, 0.8%) still had mild anaemia, including 1 (1/241, 0.4%) in the GH group and 2 (2/132, 1.5%) in the EH group. No patients had leukopenia, thrombocytopenia, hyper- bilirubinemia, pancytopenia, or a high PT time 6 months after the operation. There were no significant differences in liver function among the patient groups. No new hepatic haemangioma was found by ultrasonography.

## Discussion

Haemangioma is a benign tumour of the liver that is usually incidentally diagnosed. The majority of these patients are asymptomatic and require no treatment because the tumours are benign, have no hazardous effects and do not cause damage to other organs. However, with increasing size, large haemangiomas may be more frequently associated with symptoms and contribute to the possibility of organ compression. Symptomatic hepatic haemangioma is still a widely accepted surgical indication [11]. The risks and benefits of surgery should be carefully weighed before hepatic haemangioma resection [14].

Our study found significant differences between the GH and EH groups regarding the rates of leukopenia (4.5% vs. 10.6%), anaemia (15.7 vs. 26.5%), and thrombocytopenia (6.2% vs. 13.6%) and prolonged prothrombin time (0.4% vs. 4.5%). Correlations were found between haemangioma size, abnormalities of the haematological system and coagulation system, and liver function, which may increase the difficulty and risk of the operation. Our data suggest that enormous haemangiomas have more serious effects on the haematological system, coagulation system, and operative interventions than giant haemangiomas. Therefore, it may be necessary to reformulate the diameter standards of haemangiomas and reclassify their subgroups according to the tumour characteristics for clinical treatment.

Liver resection and enucleation are considered curative treatment options. Enucleation of hepatic haemangiomas is superior to liver resection because it is associated with reduced bleeding, shorter operative times, fewer complications and shorter hospital stays [1, 15]. In our study, although significant differences were found in postoperative laboratory indexes, operative time and occlusion time in the GH group, we found no differences in the use of the Pringle manoeuvre or the rate of blood transfusion, morbidity, mortality or postoperative hospital stay between the two types of surgery. These data indicate that the short-term outcomes of different surgical approaches are comparable, and enucleation or liver resection can be conducted safely following our decision model, as reported in other studies [16–18].

Other therapies for hepatic haemangiomas concluded: liver transplant, laparoscopic technology, transarterial embolization of the feeding artery (TAE) and radiofrequency ablation. (1) Orthotopic liver transplant represents an alternative treatment option in selected cases, with excellent outcomes in terms of safety and survival. Now, liver transplantation can be considered as a treatment option for patients with huge hemangiomas when other treatment options are not indicated or present with life-threatening conditions [19–21]. (2) Laparoscopic technology has been widely used in hepatic hemangioma, but the relevant surgical experience with diameters exceeding 10 cm is rare. A single center reported their experience with laparoscopic surgery in 58 patients with giant hepatic hemangioma larger than 10 cm. Hemangiomas larger than 15 cm in diameter was considered as a high-difficulty factor for surgery which may increased blood loss and duration of interruption, as well as hospital stays. (3) With the development of interventional radiology and improvements in catheters and superselective catheterization techniques, TAE has become a very valid option. In previous reports, TAE was shown to effectively shrink the tumor, allowing for an easier resection. The use of TAE for the treatment of giant hepatic hemangioma has the advantages of minimal trauma, few complications and good efficacy, especially in patients with high surgical risk [22]. However, TAE is still characterized by high recurrence rate and high mobidity, which should be carefully selected. (4) In recent 10 years, radiofrequency ablation (RFA) has been widely used in the treatment of hepatic hemangioma, showing advantages such as accurate, safe, minimally invasive, short hospital stay, and low cost. Although thermal ablation for giant hepatic hemangiomas can achieve satisfactory results, the complication rate is too high, especially for hemangiomas larger than 10 cm in diameter [23].

Haemorrhage remains the most important concern in the surgical management of giant hepatic haemangiomas due to the attachment of many vascular structures adjacent to the haemangioma [5]. Our study showed that the operation time was longer, and blood loss and blood transfusion were more common in the EH group. In addition, morbidity was higher in the EH group than in the GH group. These results suggest that when the diameter of a haemangioma increases, operative treatment becomes more complex, and there is a greater risk. Some scholars have also reported that operation time and blood loss significantly increase with haemangioma diameter [2, 7]. This finding was also supported by Singh [24], who found that a larger haemangioma diameter increased the difficulty of the operation. Yoshimizu previously reported that patients with larger tumours required more blood transfusions and had higher rates of postoperative complications and mortality [25].

Here are some of our experiences in reducing surgical bleeding. First, when dealing with large vessels, the surgeon should try to clamp the main hepatic vein, if possible, to reduce blood flow back to the liver and prevent air embolism. When tears occur in hepatic veins, the preferred treatment method is compression with gauze to stop the bleeding, followed by suturing with 5–0 absorbable sutures, which are reliable and safe. Second, in the process of hepatic haemangioma resection, separation should be performed along the boundary between the haemangioma and normal liver tissue to prevent damage to the capsule and reduce bleeding. Normal liver parenchyma was avoided as much as possible to reduce postoperative bile leakage. Third, surgery for central haemangiomas is more technically complex than peripheral haemangiomas. The best treatment should be standard lobectomy or embolisation when haemangiomas are deeply embedded in the liver parenchyma.

The common postoperative complications for giant hepatic haemangioma were bleeding (8 patients, 2.14%), abdominal abscesses (11, 2.95%), pleural effusion (12, 3.22%), and biliary fistula or leakage (1, 0.27%). Our study is consistent with the report of Gao [26]; the morbidity rate was 10% to 27%, and the mortality rate was 0% to 2% in patients undergoing surgery for hepatic haemangioma.

Based on the multiple logistic regression analysis, the size of the haemangioma was an independent risk factor for complications, and a larger haemangioma indicates a greater risk of complications during surgery. Jiang analysed the clinical data of 14 patients with hepatic haemangioma whose diameter was larger than 20 cm, and their postoperative complication rate was 21.4% [27], which was consistent with our results. Based on our experience, we believe that the main reasons for this are as follows. First, hepatic haemangiomas larger than 10 cm in diameter often compress the hilar blood vessels and the posterior inferior hepatic vena cava, making it challenging to apply blood flow control technology during surgery. Meanwhile, the severely compressed surgical space makes intraoperative anatomical operation very difficult. Second, large-scale hepatectomy is often required to completely remove the lesion. The patients’ postoperative vascular bed volume is reduced, and acute portal hypertension quickly occurs, which causes liver sections to easily bleed after surgery, which is also an important explanation for the high rate of postoperative complications of large hepatic haemangioma.

There are several limitations to our study. First, although our study contains large samples, the number is still insufficient, and more samples are needed to confirm our conclusions. Second, we will classify giant haemangiomas into more detailed groups according to their diameters, analyse the differences in laboratory indicators and clinical differences between them, and analyse their surgical safety and efficacy, creating a solid theoretical foundation for grouping hepatic haemangiomas.

## Conclusions

Haemangiomas with a larger size are more likely to result in haematologic and coagulation disorders. Liver resection or enucleation can be performed safely for haemangiomas ≥ 10 cm. However, haemangiomas with a diameter greater than 15 cm require a longer operation time and bleed more than those with a diameter greater than 10 cm. In addition, an increased haemangioma diameter is also associated with the amount of bleeding and postoperative complications. It is now necessary to reclassify the subgroups of haemangiomas according to tumour diameter before choosing a clinical treatment [28].

To the best of our knowledge, this is the most extensive report of giant liver cavernous haemangiomas.

## Data Availability

Data are available upon reasonable request. All data relevant to the study are included in the article or uploaded as supplementary information. All data are fully available without restriction from the corresponding author at luokunl2011@126.com.
